# What's Happened to Italian Adolescents During the COVID-19 Pandemic? A Preliminary Study on Symptoms, Problematic Social Media Usage, and Attachment: Relationships and Differences With Pre-pandemic Peers

**DOI:** 10.3389/fpsyt.2021.590543

**Published:** 2021-04-27

**Authors:** Stefania Muzi, Alessandra Sansò, Cecilia Serena Pace

**Affiliations:** Department of Education Sciences, School of Social Sciences, University of Genoa, Genoa, Italy

**Keywords:** COVID, pandemic (COVID-19), adolescents, emotional-behavioral symptoms, Binge-Eating disorder, attachment representations, risk assessment, social media disorder scale

## Abstract

Italian adolescents were confined at home for 3 months due to the COVID-19 pandemic, which exposed them to feelings of fear, uncertainty, and loneliness that may have increased their vulnerability to emotional-behavioral symptoms (e.g., anxiety) and binge-eating attitudes. Potential risk factors for these psychopathological symptoms are problematic social media usage and attachment insecurity. Therefore, this study aimed: (1) to assess emotional-behavioral symptoms, binge eating, problematic social media usage, and attachment representations of adolescents during the pandemic, comparing them with prepandemic similar samples; (2) to investigate relationships among variables, exploring the role of problematic social media usage and insecure attachment as risk factors for more psychopathological symptoms. *Participants* were 62 community adolescents aged 12–17 years, enrolled through schools, and assessed online through the following *measures:* Youth Self-Report for emotional-behavioral problems, Binge-Eating Scale for binge eating, Social Media Disorder Scale for problematic social media usage, and the Friends and Family Interview for attachment. The main *results* were: (1) 9.4% of adolescents showed clinical rates of emotional-behavioral symptoms and 4.8% of binge eating attitudes. The comparison with pre-pandemic samples revealed that pandemic teenagers showed lower internalizing, but higher other problems (e.g., binge drinking, self-destructive behaviors) and more problematic social media usage than pre-pandemic peers. No differences in binge-eating attitudes and attachment were revealed (76% secure classifications). (2) Problematic social media usage was related to more binge eating and emotional-behavioral problems, predicting 5.4% of both delinquent and attention problems. Attachment disorganization predicted 16.5% of internalizing problems, somatic complaints, and social and identity-related problems. In *conclusion*, confinement did not increase adolescents' internalizing symptoms -i.e., vulnerability to mood disorders of an anxious-depressive type- which even decreased. However, teenagers may have expressed their discomfort through other problems and symptoms of social media disorder. Further studies should explore the role of adolescents' problematic social media usage and attachment insecurity as risk factors for additional psychopathological symptoms.

## Introduction

Since December 2019, the new form of coronavirus named SARS-CoV-2 has quickly spread worldwide, and one of the most affected countries was Italy, registering more registering more than 2.85 million cases and over 96,000 deaths from late January 2020 to February 2021.

As a consequence of the growing number of cases that the health system was struggling to support, from February 2020, Italy activated legislative lockdown measures in affected regions, such as closing schools and commercial activities, travel restrictions or prohibitions, and quarantine for locals. Since March 11, when the World Health Organization (WHO) declared SARS-CoV-2 a pandemic emergency (i.e., COVID-19 pandemic), the Italian government extended the restrictions to the whole country, and all Italians were confined to their homes up to mid-May.

The exposure to this abnormal pandemic event may have negatively influenced the social, physical, and psychological functioning of children and adolescents in different ways, due to prolonged exposure to feelings of fear and uncertainty (e.g., loss of some relatives), and to physical and social isolation due to confinement (1, 2).

Indeed, international studies report that teenagers showed an increase in *emotional-behavioral symptoms* during the pandemic, especially of anxiety, depression, post-traumatic stress, and attentional problems (1, 3–9). Moreover, increased anxiety, distress, and sense of loneliness, together with the limited chance to move and buy daily fresh food, may have had adverse effects on adolescent eating behaviors, predisposing them to rely upon food as a source of comfort and an emotional regulator, which could increase their risk to engage in *binge eating attitudes* (10, 11).

By contrast, in the Co-SPACE study (12), parents reported a decrease in emotional difficulties in their teenagers, who did not recognize changes in their emotional-behavioral symptoms. Furthermore, Italian adolescents showed moderate anxiety, less than peers in other Mediterranean countries, and more healthy dietary habits during confinement (13–15).

Therefore, especially in Italian teenagers, there is a clear need for more data to define the extent of adolescents' emotional-behavioral symptoms and binge eating attitudes during this anomalous situation, together with more information on the effects of established risk or resilience factors for them.

During the lockdown, there was a dramatic increase in using social media, namely social networking sites (e.g., Facebook, Instagram, TikTok), messenger platforms (e.g., Whatsapp, Skype), and online video games that adolescents used to communicate with relatives and friends or for entertainment (16, 17). Along with concern about the pandemic, this increase was associated with adolescents' compulsive internet use, indicative of a deeper *problematic social media usage* (18). The assessment of the problematic use of social media in adolescents is relevant in preventive and clinical terms, because it can be a prodromal indicator or aggravate toward the development of a Social Media Disorder, characterized by symptoms of compulsion, tolerance, salience, and withdrawal applied to social media (19). Moreover, individuals manifesting problematic social media usage may also show desire for constant connectivity and a preference for online social interaction that lead to distress and impairments in the social and emotional functioning of the user (20–25). Moreover, pre-pandemic reviews and meta-analyses have established problematic social media usage as a risk factor for more anxious-depressive symptoms and disordered eating behaviors of adolescents (22, 26, 27). During the confinement, social media was a potential source of inaccurate or misleading health news regarding COVID-19, further predisposing users to panic distress and anxious-depressive symptoms (28–31). In addition, teenagers who used more social media during the pandemic reported more anxiety, depression, and other problems, such as self-destructive symptoms (32, 33).

On the other side, some studies suggest social media usage as helpful, rather than problematic, to cope with potential feelings of anxiety and loneliness due to the temporary restriction of face-to-face contacts at school and with friends (34–37). Therefore, there is no agreement on the risk or buffering effect of social media usage on adolescents' symptoms during the pandemic.

Another factor deserving attention is *attachment*, in terms of attachment Internal Working Models [IWMs; (38, 39)], i.e., mental representations of self, significant other, and relationships between self and significant other, stemming from early interactions with primary caregivers and generalized to become a template to further relationships. According to Bowlby (39), IWMs tend to stability across life events, influencing the social adjustment and the psychopathological vulnerability of the individual. Indeed, Rajkumar (40) employs attachment theory to understand individuals' responses and mental health outcomes during the COVID-19 pandemic, and Steele (41) specifically suggested that attachment security moderated the experience of fear during the pandemic, predisposing to more positive reactions.

By definition, a securely-attached person appears flexible and balanced in seeking closeness and separation within significant attachment relationships, valuing them as something useful and rewarding. *Securely-attached* individuals openly discuss personal attachment experiences and related emotions even if they were harsh and painful, and they typically show a desire to connect with others, which helps their lifelong social adaptation (39, 42, 43). During adolescence, securely-attached teenagers usually show better social skills, self-esteem, and adaptive stance in stressful situations, as they are more prone to seek comfort and help from parents and friends (44–46).

By comparison, *insecurely-attached* individuals usually show difficulties in relationships due to attachment strategies that prompt imbalances toward the search for excessive separation, i.e., insecure-dismissing, or unwarranted closeness, i.e., attachment preoccupation (42, 45). On the one hand, individuals classified as *insecure-dismissing* appear unbalanced toward exploration; they tend not to seek attachment figures for help or comfort, preferring to rely on themselves and portraying themselves as strong, normal, and independent. Insecure-dismissing individuals recount their attachment experiences untruthfully, showing idealization or derogation of the attachment figures and the tendency to normalize the negative experiences (42). Overall, they show an affective hypo-activation in response to emotions and stimuli from significant relationships and excessive attention to the relationships' instrumental and concrete aspects.

On the other hand, individuals classified as *insecure-preoccupied* prefer proximity to the detriment of exploration, showing anxious hypervigilance toward attachment figures and signals coming from attachment relationships. Such hyper-evaluation of attachment relationships may lead to an excessive response of anger or passivity toward parents or an age-inappropriate desire to please or substitute them, i.e., role-reversal. Both types of insecure patterns predispose an individual to low adaptive responses to stressors, especially if coming from attachment relationships since when insecurely-attached individuals are troubled or need help, they find it more challenging to seek comfort or help from others and to regulate their negative emotions (45, 47, 48).

Lastly, individuals classified as disorganized simultaneously manifest contradictory attachment strategies (e.g., dismissing and preoccupied) or no strategy structuring individual's relational behavior and expectations on the others' one, often because of previous unresolved frightening and traumatic relational experiences (42).

Pre-pandemic literature highlighted that teenagers showing insecure and disorganized attachment IWMs are more at risk for both emotional-behavioral symptoms and binge-eating attitudes, and they also engage a more problematic social media usage, potentially increasing its negative effect on teenagers' symptoms (49–54).

During the pandemic, a single study on attachment was published demonstrating Italian adults who showed the worst mental health outcomes with an insecure-preoccupied pattern, while securely-attached adults showed better mental health (55).

Given the above, securely-attached teenagers may have been more resilient in facing a fearful situation (such as the COVID-19 pandemic and consequent restrictions), thanks to their secure IWMs, which enabled them to be more capable of managing feelings of fear and anxiety or maintaining positive interactions with friends despite the unusual online form. However, no published studies explored teenagers' attachment during the pandemic.

Furthermore, no research simultaneously explored the role of teenagers' problematic social media usage and attachment IWMs on their symptoms during the lockdown due to the COVID-19 pandemic. Therefore, the current study had two *aims*:

(a) To assess emotional-behavioral and binge eating symptoms, problematic social media usage (i.e., social media disorder symptoms), and attachment insecurity in community adolescents during the pandemic, comparing assessments of community adolescents before the pandemic (from similar groups). Higher symptoms in pandemic participants than pre-pandemic groups were hypothesized, except for attachment, where no difference was hypothesized.
(b) To examine the relations among emotional-behavioral symptoms and binge-eating attitudes with problematic social media usage and attachment IWMs of community adolescents during the pandemic. More emotional-behavioral symptoms and binge-eating attitudes in teenagers with more problematic social media usage and/or higher attachment insecurity/disorganization were hypothesized.

## Materials and Methods

### Participants and Procedure

Sixty-two community adolescents aged 12–17 years [Mean (M) = 15.43, Standard Deviation (SD) = 1.65, 37% boys] were enrolled from late March to early May 2020, through schools from Northern Italy for more extensive longitudinal research. Participants were included in the current pilot comparative study if they: 1. were between 12 and 17 years of age; 2. had no diagnosis for severe psychiatric disorders or intellectual or physical disability; 3. have been assessed during the lockdown, between April and May 2020.

All participants eligible for this study accepted to participate (0% attrition), and the majority of them attended high school (73%, *n* = 43), while 30% of them (*n* = 19) attended middle schools. Most of them came from intact families (82%) and had at least one sibling (55%) or more (18.4%). The mean age of adolescents' mothers was 48.70 years (SD = 4.70), while the mean age of adolescents' fathers was 51.20 (SD = 6.16), and almost all parents achieved high school diplomas or higher levels of education and had a job (99%).

Pre-pandemic groups for the comparison were drawn by Marino et al. (23), Pace and Muzi (10), and Pace et al. (56) because the community adolescents from these studies were similar to participants, but their assessments were carried out before the pandemic (between 2019 and January 2020). Indeed, adolescents in these studies lived in Northern Italy and in low-risk intact families, similarly than participants. The group from Pace and Muzi [(10); *N* = 382, age-range 13–18 years, M = 15.59, SD = 1.10, 39% boys] came from the same region and similar social-familiar background than the current one, showing no differences in age, *t*(442) = 0.98, *p* = 0.320 (95% CI 0.48–0.16), or gender distribution, a χ^2^ (1) = 0.08, *p* = 0.770, φ = 0.02 (95% CI 0.61–1.92). This group was compared on prevalence and scores for *binge eating attitudes* and for *emotional-behavioral symptoms* (using unpublished data) because the Italian validation study (57) did not report the prevalence and mean scores of the normative Italian sample in the Youth Self Report [YSR; (58)] here used. The group from Pace et al. (56); *N* = 110, age-range 11–17 years, M = 14.22, SD = 1.84, 50% boys) also came from the same region and had similar social-familiar backgrounds of participants, showing no difference in gender distribution, χ^2^(1) = 2.54, *p* = 0.120 φ = −0.11 (95% CI 0.36–1.12), but they were younger than participants, *t*(170) = 4.29, *p* = 0.001 (95% CI 0.65–1.77). Given this study employed the Friends and Family Interview [FFI; (45)], this pre-pandemic group was selected because this was the largest group of Italian community teenagers assessed with the same instrument. Lastly, the group from Marino et al. [(23); *N* = 761, age-range 13–19 years, M = 15.49, SD = 1.03; 56.5% boys] came from the Northern area of the country, showing no difference in age with participants, *t*(821) =0.41, *p* = 0.610 (95% CI −0.34 to 0.22), while the gender distribution differs due to more girls in the current study, a χ^2^(1) = 6.14, *p* = 0.013, φ = −0.18 (95% CI 0.28–0.86. This group was selected for the comparison on social media usage scores because it is the only pre-pandemic group of Italian teenagers assessed with the questionnaire used in this study, the Social Media Disorder Scale [SMDS; (19)].

The research received approval (protocol n. 037) from the University Ethical Committee of the Department of Educational Sciences of the University of Genoa in Italy, which approved research procedure and purposes, as in line with the international research's broader ethical criteria community (Declaration of Helsinki). During online class hours, the research team informed potential participants and their families about the research aims and procedures, reminding them that participation was voluntary and explaining the rights in the informed consent sheet (privacy, withdrawal from the research without motivating the choice, etc.). After that, all legal caretakers of adolescents who agreed to participate signed an informed consent *via* an electronic signature. Thus, participants filled online questionnaires *via* Lime-survey software and trained M.A. students in psychology interviewed them through taped video calls *via* Skype in compliance with social distancing rules during the pandemic. Participants filled all questionnaires on an online form accessible through a personal secret code, which the research group used to trace the questionnaire-participant combination.

Given the preliminary nature of this study, data of FFI attachment interviews were available for 29 participants (47%), since the coding of the remaining 33 was in progress at the time of this submission. The subgroup of participants with FFI (*n* = 29, M_age_ = 14.80, SD = 1.80, 40% boys) did not significantly differ from the entire sample in age, gender distribution, family structure, or number of siblings, all *p* > 0.05.

### Measures

*The Youth Self-Report 11–18* [YSR; (57, 58)] is a 112-item self-report questionnaire to measure emotional-behavioral symptoms in children and adolescents aged 11–18 years old. The adolescent rates his/her symptoms in the previous 6 months on a three-point Likert-type scale (0 = not true, 1 = sometimes true, 2 = often true). Scores are assigned to several syndromes grouped in the scales: internalizing problems (includes withdrawal/depression, anxiety, somatic complaints), externalizing problems (includes aggressive behaviors and delinquency), other problems (includes social problems such as binge drinking, attention problems, thought problems such as suicidality or dissociative symptoms, and identity problems, such as gender-related and self-destructive problems). There is also a global score on the scale of the total problems. The YSR showed good internal validity (Cronbach's α: 0.71–0.95) and good test-retest reliability (*r* = 0.68). In this study, Cronbach's α was 0.93.

The *Binge Eating Scale* [BES; (59, 60)] is a widely known 16-item self-report questionnaire specifically designed to evaluate the presence and frequency of binge-eating symptoms. There are three or four statements for each item, and the person indicates which statement reflects more his/her condition (e.g., item 1 has “I worry about my appearance, but that doesn't normally make me dissatisfied with myself” as B alternative). Each statement corresponds to a score from 0 to 3, sometimes with two options scoring three points in the same item, e.g., items 3 and 7. The final score range from 0 to 46, and higher scores indicate more severe binge-eating attitudes. Three thresholds constitute cut-off scores: <17 no risk of binge eating, >17 moderate binge eating; > 27 clinical risk of binge eating, a Binge Eating Disorder diagnosis is warranted. The BES shows good reliability (Cronbach's α = 0.87), and Cronbach's α was 0.81 in this study.

The *Social Media Disorder Scale – 9 items* [SMDS; (19); Italian version (23)] is a short version of an extended 27-item version to capture the problematic usage of social media (Whatsapp, Facebook, etc.) during the past year. The person provides yes/no answers to different questions (e.g., “In the last year, did you realize you were sick when you couldn't use social media?”), which cover nine problematic criteria: persistence, escape, conflict, displacement, deception, withdrawal, preoccupation, tolerance, and problems. In the version used in this study, the SMDS showed acceptable reliability (Cronbach's α = 0.76), and the same value (0.76) was found in this study.

The Friends and Family Interview - COVID-19 version [FFI; (45); Italian version (56)] is an audio- or videotaped age-adapted semi-structured interview to assess attachment representations in children and adolescents from 10 to 17 years of age. The child is asked about different relationships with potential attachment figures in adolescence, such as parents, but also friends, sibling(s), and at school. The FFI coding system is based on the Adult Attachment Interview's one (42), but it is different because the FFI allows both a categorical and a dimensional assessment of the attachment. Indeed, scores (0–4 points) are assigned on several scales in different domains (coherence, reflective functioning, self-esteem, relationship with the best friend and with siblings, affective regulation strategies, and differentiation of parental representations), and on the basis of these scores, the rater assigns a score on each scale for the attachment patterns, corresponding to the traditional four categories: *Secure-Autonomous* (S/F), indicative of a coherent narrative, where the person shows flexibility, the capacity to need and miss others, the value of attachment relationships, high adaptive response, and little or no psychological defenses; *Insecure-Dismissing* (Ds), indicative of poorly coherent narratives due to minimization of the attachment, idealization, or derogation of the self or the attachment figures, and excessive attention to instrumental aspects of the relationships; *Insecure-Preoccupied* (E), indicative of low coherence and inflexibility due to anxious hypervigilance within attachment relationships and age-inappropriate responses of anger, role reversal or passivity in discussing attachment experiences with parents; *Insecure-Disorganized* (D) when the incoherent narrative reflects the lack of an organized attachment strategy or simultaneous incompatible and contradictory strategies (e.g., Ds and P) and the presence of bizarre or frightening content in the IWMs that derives from potentially traumatic experiences, which seems unresolved. The best-fit attachment category is assigned based on the higher score in the four scales for such attachment patterns. The last version [V5, (61)] used in this study contains two additional questions to investigate the effects of the pandemic on the attachment relationships (“Due to the current situation of the pandemic emergency, have you experienced the loss of people important for you (relatives, e.g., grandfather, friends)?,” “Due to the current situation of the pandemic emergency, have there been changes in the relationship with your parents?”), added in agreement with the author H. Steele.

In this study, all interviews were videotaped and transcribed verbatim, covering all the names of people, animals, and places to ensure the participants' privacy. All interviews were coded by two independent certified reliable coders (the first and the last author), with 94% agreement on both secure-insecure and four-way classifications (*k* = 0.86, *p* < 0.001) and 100% on organized-disorganized ones (*k* = 1, *p* < 0.001). A third independent certified coder resolved any disagreements between the two coders. Pearson interrater correlations on FFI pattern scales were all significant (all *p* < 0.001), and the final score used in this study was the average scores between those assigned by the first and second rater.

The FFI shows good psychometric proprieties (56, 62–65). Cronbach's α for the reliability was 0.74 in this study.

### Analytic Plan

SPSS version 25 has been used to perform all statistical analyses, and the results were considered statistically significant with *p* < 0.05, reporting 95% Confidence Intervals [CI] when appropriate. All analyses inclusive of questionnaires were performed on the whole sample (*N* = 62), while analyses inclusive of the FFI were performed on the subgroup with data available (*n* = 29), already checked as being homogeneous with the larger group (see above, participants' section).

According to cut-off scores in the literature, prevalence rates were defined, considering the *T* scores for the YSR and the raw scores for the BES. The chi-square test was employed to compare pandemic and pre-pandemic groups on the percentage distribution of attachment categories in the FFI (56), reporting Cramer's phi (φ) and Odds ratio [OR] as measures of effect size. The *t*-test was used to an initial control for gender's effect, by comparing scores of boys and girls in all study variables; then, pandemic and pre-pandemic Italian groups on YSR, BES, SMDS, and FFI pattern scores, reporting descriptive statistics (M and SD) and group size for all comparisons groups [(10, 23, 56) see Participants and Table 1].

**Table 1:** Comparison of scores for emotional-behavioral symptoms^a^, binge-eating attitudes^b^, problematic social media usage^c^, and attachment representations^d^ between 62 Italian community adolescents during the COVID-19 pandemic and similar pre-pandemic samples^e^.

	**Pandemic adolescents**		**Pre-pandemic adolescents**		***t*_**(df)**_**	***p***	**95% CI**	
	**M**	**SD**	**M**	**SD**			**LL**	**UL**
YSR/total problems	60.89	25.48	68.10	25.46	2.07 _(442)_	**0.040**	−4.06	0.36
YSR/internalizing problems	10.94	6.76	17.81	10.22	5.11 _(442)_	**0.000**	−9.51	−4.23
YSR/externalizing problems	12.23	6.97	12.09	7.32	0.14 _(442)_	0.880	−1.82	2.10
YSR/other problems	12.81	5.54	8.78	4.93	5.86 _(442)_	**0.001**	2.68	5.38
BES/total score	6.00	5.90	6.40	5.70	0.51_(44)_	0.610	−1.97	1.14
SMDS/total score	2.47	2.24	1.65	0.53	7.8 _(821)_	**0.000**	0.61	1.02
FFI/secure-autonomous	3.08	0.63	2.85	0.80	1.19 _(127)_	0.230	−0.15	0.61
FFI/insecure-dismissing	1.84	0.93	1.80	0.82	1.90 _(127)_	0.841	−0.37	0.45
FFI/insecure-preoccupied	1.32	0.47	1.42	0.58	0.71 _(127)_	0.482	−0.38	0.17
FFI/insecure-disorganized	1.00	0.00	1.14	0.39	1.55 _(127)_	0.121	−0.32	0.04

a *Youth Self Report 11–18 years (YSR)*.

b *Binge Eating Scale (BES)*.

c *Social Media Disorder Scale (SMDS)*.

d *Friends and Family Interview (FFI), n = 29*.

e *YSR and BES (10), N = 382, M_age_ = 15.6; SMDS (23), N = 761, M_age_= 15.5; FFI (56), N = 110, M_age_ = 14.2. Significant values are highlighted in bold*.

The Pearson's *r* correlation coefficient was employed to preliminarily check the age's effect on all study variables and explore relationships among them. General linear models were performed to examine the role of problematic social media usage and attachment patterns on participants' scores for emotional-behavioral symptoms and binge-eating attitudes, reporting partial eta squared (η^2^) and observed power.

## Results

### Preliminary Analyses

Girls and boys did not show differences in YSR, BES, SMDS, and FFI pattern scores, all *p* > 0.191. In line with the literature, older adolescents showed more internalizing problems, *r* = −0.273, *p* = 0.032, specifically more anxiety, *r* = −0.253, *p* = 0.047.

### Prevalence and Differences With Pre-pandemic Adolescents in Emotional-Behavioral Symptoms, Binge-Eating Attitudes, Problematic Social Media Usage, and Attachment Representations

Regarding emotional-behavioral symptoms, the t-scores of six participants (9.4%) exceeded the cut-off t-scores for clinical risk in each category of total, internalizing and externalizing problems.

In terms of the prevalence of *binge eating*, three participants (4.8%) were at risk for moderate binge eating, and none showed clinical risk.

Table 1 shows all comparisons between pandemic and pre-pandemic adolescents.

As shown in Table 1, contrary to expectations, pandemic adolescents showed lower levels of emotional-behavioral problems than their pre-pandemic peers, specifically less internalizing ones. However, the pandemic group showed significantly more other problems.

Concerning binge-eating attitudes, pandemic and pre-pandemic adolescents demonstrated no differences in BES scores, which did not confirm the hypothesis.

As hypothesized, pandemic adolescents revealed a more problematic social media usage than pre-pandemic peers.

Lastly, in the subgroup with FFI (*n* = 29), the distribution of attachment categories was 22 (76%) secure and 7 (24%) insecure, all insecure-dismissing, while no participants received insecure-preoccupied or disorganized classifications. The chi-square comparison with the pre-pandemic group (56) revealed no differences in the percentage distribution of secure-insecure classifications, χ^2^(1) = 1.99, *p* =0.161, φ =0.1 (OR = 1.56, 95% CI 0.84–2.90). As shown in Table 1, the pandemic group did not show statistically significant differences with the pre-pandemic group even in the FFI attachment patterns' average scores.

### Relationships Among Study Variables

Table 2 shows correlations among study variables, confirming some expected between more problematic social media usage and higher total score of emotional-behavioral symptoms and more binge-eating attitudes. Further, attachment insecurity scales for preoccupation and disorganization showed relations with more internalizing and other problems.

**Table 2:** Pearson's correlations between scores of emotional-behavioral symptoms^a^ and binge-eating attitudes^b^ with problematic social media usage^c^ and attachment patterns^d^ in 62 Italian community adolescents during the COVID-19 pandemic.

	**SMDS**	**FFI/Secure-autonomous**	**FFI/Insecure-dismissing**	**FFI/Insecure-preoccupied**	**FFI/Insecure-disorganized**
YSR/total problems	0.23^*^	−0.04	−0.08	0.106	0.27
YSR/internalizing problems	0.19	−0.03	−0.09	0.48^*^	0.37^*^
Withdrawal/depression	0.21^*^	−0.25	0.10	0.01	0.08
Anxiety	0.24^*^	−0.12	0.02	0.17^*^	0.28
Somatic complaints	0.03	0.07	−0.10	0.05	0.44^**^
YSR/externalizing problems	0.24^*^	0.06	−0.21	0.07	0.19
Aggressive behaviors	0.19	0.08	−0.19	0.15	0.20
Delinquency	0.28^*^	0.03	−0.20	−0.06	0.17
YSR/other problems	0.18	−0.02	−0.00	−0.03	0.13
Social problems (e.g., binge drinking)	0.24^*^	−0.07	0.05	0.22	0.38^*^
Thought problems (e.g., suicidality)	0.26^*^	−0.15	−0.02	0.01	0.07
Attention problems	0.27^*^	−0.04	−0.08	0.12	0.12
Identity-related problems (e.g., self-destructive)	0.20	−0.23	0.15	−0.01	0.42^*^
BES/total	0.23^*^	0.16	−0.17	−0.12	−0.18

* *p < 0.05*.

** *p < 0.01*.

a *Youth Self Report 11–18 years (YSR)*.

b *Binge Eating Scale (BES)*.

c *Social Media Disorder Scale (SMDS)*.

d *Friends and Family Interview (FFI), n = 29*.

### Models of Risk Prediction

Given the correlations, the role of *problematic social media usage* was explored as a potential predictor of more total and externalizing problems and binge-eating attitudes, but no predictive model was statistically significant, all *p* < 0.076.

Problematic social media usage predicted 5.4% more delinquent behaviors, *F*_(1, 60)_ = 4.34, *p* = 0.041, adj. *R*^2^ = 0.054 (95% CI 0.01–0.65), η^2^ = 0.08, Observed Power = 0.59; and 5.4% more attention problems, *F*_(1, 60)_ = 4.3, *p* = 0.050, adj. *R*^2^ = 0.054 (95% CI 0.01–0.75), η^2^ = 0.07, Observed Power = 0.58.

Concerning the role of attachment, participant's age, the *insecure-preoccupied* and *insecure-disorganized* patterns were entered as predictors of internalizing problems. The final model explained 16.5% of the variance in internalizing problems' scores, *F*_(1, 28)_ = 2.84, *p* = 0.05, adj. *R*^2^ = 0.165 (95% CI −34.2 to 36.1), η^2^ = 0.00, Observed Power = 0.05. The analysis of β coefficients revealed the disorganized pattern as a unique significant predictor, *p* = 0.040. Specifically, attachment disorganization was the unique predictor for 17% more somatic complaints, *F*_(1, 28)_ = 6.68, *p* = 0.015, adj. *R*^2^ = 0.169 (95% CI −17.9 to 0.27), η^2^ = 0.20, Observed Power = 0.70. Moreover, attachment disorganization was the unique predictor for 11% more social problems, *F*_(1, 28)_ = 4.50, *p* = 0.043, adj. *R*^2^ = 0.111 (95% CI −11.9 to 1.5), η^2^ = 0.143, Observed Power = 0.534, and 14.5% more identity-related problems, *F*_(1, 28)_ = 33.94, *p* = 0.024, adj. *R*^2^ = 0.145 (95% CI −17.1 to 22), η^2^ = 0.17, Observed Power = 0.64.

Finally, the model with predictor's age and *attachment preoccupation* allowed for the prediction for 15.2% of the variance in anxiety scores, *F*_(1, 28)_ = 4.23, *p* = 0.050, η^2^ = 0.07, Observed Power = 0.26, with no independent significant predictors.

## Discussion

In this study, Italian community adolescents have been assessed in emotional-behavioral and binge eating symptoms, problematic social media usage, and attachment representations while confined at home due to the COVID-19 pandemic.

An abnormal event such as a pandemic, characterized by prolonged feelings of fear and uncertainty, was expected to increase adolescents' psychopathological vulnerability. Therefore, in this study, the first aim was to compare the adolescents' prevalence and/or levels of symptoms with similar groups before the pandemic, but most of the results have not confirmed the hypotheses.

From the observation of the prevalence data, the pandemic group did not show more *emotional-behavioral symptoms* than pre-pandemic peers, respectively 9.4 vs. 8.2% in the parent-reported normative sample (57). On the contrary, the comparison with the scores of a similar and more recent group (10) showed that adolescents confined to home showed less emotional difficulties, specifically fewer internalizing symptoms than pre-pandemic peers. Therefore, the vulnerability of these adolescents to anxious-depressive symptoms and somatic complaints may not have increased during the pandemic, as expected based on the literature (1, 3–6, 8, 9). On the contrary, the emotional-behavioral difficulties of an anxious-depressive type seem to have decreased, in line with the Co-SPACE study (12) findings. These results also align with those of national studies, where Italian teenagers appeared only moderately anxious, different from teenagers in international studies (14, 15, 34). Perhaps this possible reduction of internalizing symptoms was favored by the slowing down of school rhythms and the blocking of extracurricular activities, which constitute two significant stressors at the basis of anxiety symptoms in adolescence (5, 66). Moreover, contrary to expectations, adolescents probably did not respond to negative feelings prompted by the confinement by seeking comfort in food, as they did not show more binge-eating attitudes than their pre-pandemic peers (11).

Statistical comparison was impossible to perform regarding *binge eating attitudes*, but the prevalence was slightly lower in this group than in peers in the same Italian region before the pandemic [4.8 vs. 6%, (10)]. Therefore, participants adolescents seem to have had healthy eating habits during the pandemic, and future research could investigate whether these habits have become even healthier than before the pandemic, as suggested by other national findings (13). Otherwise, the absence of unhealthy eating behaviors during confinement can be due to a beneficial effect of having home-cooked and regular meals instead of pre-packed meals in the dining-halls, as happened in pre-pandemic adolescents' lives (67).

On the other side, pandemic adolescents showed higher levels of *other problems* and a more *problematic social media usage* than peers before the pandemic.

The first result may suggest that the new generation of adolescents express their discomfort through alternative type symptoms rather than the expected “traditional” internalizing forms, as noted by other authors (10, 68, 69). Indeed, the YSR other problems scale rates social, thought, attentional and identity-related problems, which are indicative of engagement in common risky behaviors of adolescence: for instance, high scores in social problems indicate conducts of binge drinking, substance abuse, possible bullying in the form of teasing the others, or being teased by the others as a victim, and general withdrawn in social relationships. The scale for thought problems rates symptoms of suicidal ideation and identity-related problems include self-destructive behaviors, e.g., self-cutting, or confusion about gender identity. A future investigation could specify in what syndromes pandemic groups resulted more vulnerable than pre-pandemic ones, e.g., more suicidal ideation rather than gender-identity confusion, and explore possible reasons behind differences. For instance, pre-pandemic literature suggests (68) that self-destructive behaviors can be prompted by interpersonal stress, so a future investigation could explore more deeply whether online forms of communication and social distancing due to pandemic restrictions were perceived by adolescents as an interpersonal source of stress.

The second result aligns with Fernandes et al. (18), suggesting that the increase in social media use during confinement—to follow school lessons or maintain contact with relatives and friends—may have increased problematic use by Italian adolescents, who showed more symptoms of social media disorder than pre-pandemic peers (23). Adolescents' problematic social media usage was also linked to their higher emotional-behavioral symptoms and disordered eating behaviors, confirming the hypotheses based on pre-pandemic literature (27, 70). In particular, teenagers reporting more symptoms for social media disorder in SMDS were more likely to declare attention problems in the YSR, perhaps due to excessive attention focused on social media, which could affect the adolescent's ability to pay attention to other aspects of life, such as face-to-face relationships with family members and schoolwork (71). Taken together, these results seem to suggest problematic pathways associated to social media usage during the pandemic, rather than support their beneficial effect suggested by other studies (36, 37).

The other factor investigated as possibly related to adolescents' psychopathology during the pandemic was *attachment*. As expected, pandemic adolescents and similar pre-pandemic peers did not show significant difference rates of secure-insecure classifications, supporting the idea that IWMs may tend toward stability even through stressful life events, including a pandemic (39). Future longitudinal studies assessing adolescent's attachment during and after confinement can confirm this hypothesis.

Nevertheless, utilizing continuous scores on attachment patterns enables evaluations based on models of risk assessment, providing valuable information that would not have been obtained by focusing the investigation only on the “traditional” categories of attachment, as defined in the attachment literature. Indeed, it confirmed that IWMs might support or set back the adolescents' adaptation throughout the life span, as more insecurely attached adolescents showed more emotional and behavioral problems during the confinement due to COVID-19, confirming the hypothesis based on the study on Italian adults (55). In general, insecurely-attached teenagers could have been less able than secure ones to share their feelings related to the pandemic, benefiting perhaps less from the reward effects of self-disclosure (40, 72). Specifically, both attachment preoccupation and disorganization scores were related to more internalizing problems, but only the disorganized pattern was predictive of them. Attachment disorganization was also a unique predictor for broader types of syndromes, such as somatic complaints and social and identity-related problems, supporting the meta-analytical importance assigned to this pattern to increase vulnerability the psychopathology of children and adolescents (51). In this case, the frightening COVID-19 situation and the forced confinement with attachment figures within the family may have exposed adolescents lacking an organized attachment strategy to an unmanageable emotional burden (40, 41). Continued exposure to external triggers due to the pandemic and attachment-related emotions may have overwhelmed disorganized teenagers, exacerbating social problems, such as binge-drinking during video calls with friends, or self-destructive attitudes or identity issues (73, 74).

However, even if less relevant than disorganization, the higher preoccupation in attachment was predictive of more anxiety, in line with Moccia et al. (55) study and meta-analytical findings (75, 76). This suggests that adolescents who are hyper-vigilant to signs of attachment and who show age-inappropriate excessive concern for parents' well-being may have been more vulnerable to anxiety than peers with different attachment IWMs (75). If the parents still went out to go to work or go shopping during confinement, the parental outdoor activity exposed more both parents and adolescents to the risk of contracting the virus, which could have also triggered adolescent anxiety. Moreover, friends' forced distance could have sharpened the separation anxiety typical of individuals guided by a preoccupied pattern.

Overall, the current results may suggest two main conclusions: on the one hand, and contrary to expectations, the COVID-19 pandemic and the consequent confinement did not seem to have had severe consequences for the mental health of these Italian adolescents, who have not shown more psychopathological symptoms than their pre-pandemic peers. On the other hand, both the increase in problematic social media usage—prompted by confinement—and attachment insecurity have been confirmed risk factors for adolescents' symptoms, and longitudinal studies should examine their effect during and after the pandemic.

### Limitations and Future Directions

This study may be the first to assess adolescents' attachment representations during the pandemic through an age-adapted interview rather than through self-report questionnaires, which may be less sensitive in detecting insecurity and disorganization in adolescents (51). This study also assesses for the first time the adolescents' problematic social media usage during the pandemic, connecting both this aspect and teenagers' attachment with their emotional-behavioral symptoms and binge-eating attitudes. However, these preliminary results are partial and cannot be generalized due to many limitations. At first, the sample size was small since only the participants whose data were already available were selected; in particular, there was little data on teenagers' attachment due to the long time required to code interviews. This limited number of participants also affected the power of the statistical analyses performed. In this regard, a further limitation is the lack of multiple comparison correction, increasing the risk of type 1 errors.

Moreover, despite the absence of gender differences, most participants were girls, which limited the sample's representativeness. Regarding this point, participants and pre-pandemic groups selected slightly differed in gender or age, possibly reducing the magnitude of the results for the comparisons on attachment and social media usage. More extensive studies with participants more balanced for gender and age should examine the role of these demographics, as pre-pandemic literature suggest girls as more secure and older teenagers more secure in attachment, and lower problematic social media usage in boys and at older ages (23, 63).

Second, except for the FFI, all measures were questionnaires, which poses the risk of biases associated with this kind of measure's exclusive use. Third, the mutual relationships between problematic social media usage and attachment representations were not explored, despite the risk of the former being more significant in the case of attachment insecurity; thus, further exploration in this sense could help to detect their possible moderating effects on psychopathology.

Furthermore, other unexplored relationships may have moderated the results obtained, such as the effect of psychopathological symptoms on binge-eating attitudes (10), as well as the relations between the variables here examined with others not investigated, such as the social norms and coping skills, or reflective functioning, or the role of the family in the transmission of the clinical vulnerability (23, 41, 77, 78). For instance, problematic family functioning or excessive parental distress are related to more teenager's emotional-behavioral problems, and future studies can address these relations during the pandemic (69, 79).

Moreover, pre-pandemic literature suggests several subjective variables associated with those other problems where pandemic participants showed higher rates than pre-pandemic peers. Specifically, teenagers lacking in time-perspective, i.e., with poor integration of past experiences with the present self-representation, and/or lacking of a view of the future, are more likely to show thought problems in the form of suicidal ideation, especially if insecurely-attached (80). Therefore, future studies can investigate this kind of teenagers' symptoms in light of a possible “suspended” view of the future during the confinement due to the pandemic. Further, social problems such as binge drinking and substance abuse, binge eating attitudes, and problematic social media usage have been all related to adolescents' sensation-seeking, which can lead to a risky decision-taking (81–84).

Yet another element that remained unexplored is the effect of the adolescent's brain development, still in progress, on his/her perception of risk related to the pandemic, or even on his/her propensity for problematic use of social media, which future studies could examine. Lastly, a deeper discussion of the prevalence emotional-behavioral and binge eating symptoms was hidden by limited national data in the literature using the same instruments in the same age range. In this regard, the discussion of the results in light of current findings was also limited by scarce studies addressing these variables in adolescents during the pandemic. Further studies are needed to substantiate this research's results, with larger and more representative samples, possibly using mixed methods to assess all study variables.

## Data Availability Statement

The raw data supporting the conclusions of this article will be made available by the authors, without undue reservation.

## Ethics Statement

The studies involving human participants were reviewed and approved by Research Ethical Committee (CER), Department of Education Sciences, University of Genoa. Written informed consent to participate in this study was provided by the participants' legal guardian/next of kin.

## Author Contributions

All authors provided a substantial contribution to the conception and design of the study and the acquisition, analysis, interpretation of data, participated in drafting, critically revising the article, and gave final approval for the version to be submitted.

## Conflict of Interest

The authors declare that the research was conducted in the absence of any commercial or financial relationships that could be construed as a potential conflict of interest.
